# Work-related stress of companies' directors during the first lockdown due to the COVID-19

**DOI:** 10.3389/fpsyt.2022.975953

**Published:** 2022-12-22

**Authors:** Frédéric Dutheil, Carole Jeanton, Audrey Vilmant, Céline Lambert, Maelys Clinchamps, Ukadike Chris Ugbolue, Jeannot Schmidt, Guillaume T. Vallet, Jean-Baptiste Bouillon-Minois

**Affiliations:** ^1^Université Clermont Auvergne, CNRS, LaPSCo, Physiological and Psychosocial Stress, CHU Clermont-Ferrand, Preventive and Occupational Medicine, WittyFit, Clermont-Ferrand, France; ^2^Université Clermont Auvergne, CHU Clermont-Ferrand, Occupational Medicine, Clermont-Ferrand, France; ^3^Association Interentreprises en Santé au Travail La Prévention Active, Riom, France; ^4^CHU Clermont-Ferrand, Biostatistics, Clermont-Ferrand, France; ^5^School of Health and Life Sciences, Institute for Clinical Exercise and Health Science, University of the West of Scotland, Glasgow, United Kingdom; ^6^Université Clermont Auvergne, CNRS, LaPSCo, Physiological and Psychosocial Stress, CHU Clermont-Ferrand, Emergency Medicine, Clermont-Ferrand, France; ^7^Université Clermont Auvergne, CNRS, LaPSCo, Physiological and Psychosocial Stress, Clermont-Ferrand, France

**Keywords:** stress at work, company directors, lockdown, COVID-19 pandemic, occupational health

## Abstract

**Background:**

The COVID-19 pandemic and the first lockdown were particularly stressful with a major economic impact, but the impact on stress of company directors was not known. Therefore, this study aimed to assess that impact and the characteristics of companies the most at risk.

**Method:**

A online questionnaire was sent to 13,114 company. It assessed stress at work, number of employees, sector of activity, business activity rate and geographical location. It studied the mean stress levels, the percentage of stress > 8/10 and carried out an analysis of the characteristics of the most at-risk companies.

**Results:**

A total of 807 company directors responded. Their stress levels increased by 25.9% during lockdown and 28.7% of them had a stress > 8/10. Sectors which had the biggest increase in stress levels during lockdown were retail trade, wholesale trade, and nursing homes. Sectors the most at risk of stress >8/10 during lockdown tended to be nursing homes, pharmacies, and IT activities. Biggest companies had the highest increase in stress levels.

**Conclusion:**

The first lockdown of the COVID-19 pandemic had a major impact on the stress of company directors. Directors of large companies were the most exposed to stress as well as medical and IT activities.

## 1. Introduction

Stress at work is a major issue, causing both physical (1–3) and mental (4, 5) pathologies, but also with a strong economic impact. Most studies on stress at work focus on stress of employees (6), but very few on stress of company directors (7). In this context, the coronavirus disease (COVID-19) pandemic (8), with a global lockdown of half of the world's population created a particular stressful climate that also likely impacted company directors. While very few studies assessed mental health of company directors in relation to the COVID-19 pandemic, they focused on anxiety (9), depression, and burnout (10, 11), but only one assessed stress at work (12). Moreover, none of the aforementioned study retrieved the evolution of stress of company directors before, during, and after the first global lockdown. The COVID-19 outbreak severely disrupted the global economy and impacted gross domestic products (13). All economic sectors have been affected (14). Some experienced an overload of activities such as health or informatics (IT) sectors, while others had to stop such as non-essential businesses. Despite aforementioned studies searched for risk factors of mental health disorders, none evaluated the impact of economic sectors on stress levels of company directors during the COVID-19 pandemic. In all cases and whatever the number of their employees, company directors have had to adapt to new and changing operating rules and to a major impact on their economy (15). Lastly, even if some of previous studies computed regression analyses, they did not quantity the risk of stress depending on characteristics of companies. We hypothesize that a follow-up (before, during and after lockdown) will detect the most at-risk directors to begin rapid action and to build efficient preventive strategy (16).

Therefore, the main aim of this study was to assess the stress of company directors, across the first stages of the pandemic (before, during, and after the first global lockdown). Secondary aims were to study the characteristics of companies the most at-risk of stress–particularly the impact of economic sectors, and to quantify the risk of stress of company directors.

## 2. Materials and methods

### 2.1. Study design

The main occupational health department of Auvergne, France, followed 14,148 companies at the time of the first wave of the COVID-19 pandemic. An online questionnaire was sent at the beginning of July 2020 to all the company directors followed by the occupational health department of Clermont-Ferrand without any selection or randomization. We defined company director as those who are at the top of the firm. The questionnaire was sent by email, and anonymized. Those who did not provide an email address were excluded. The study was approved by the ethics committee (CPP Sud-Est VI Clermont-Ferrand) and registered on ClinicalTrials.gov number NCT04308187).

### 2.2. Outcomes

The main objective was to assess the stress of company directors using three visual analog scales over three time periods. The three VAS were the same in size and methodology. The first one was before French lockdown (i.e., before March 17, 2020), during French lockdown (i.e., between March 17 and May 10) and after French lockdown (i.e., after May 11). Visual analog scale is a validated tool used by occupational physicians to assess stress at work (17, 18). Visual analog scale (VAS) has been validated for the assessment of stress in clinical practice (8) and is currently used by occupational physicians to quickly identify the most stressed people requiring urgent action (9). The use is simple to implement, easy to understand, and quick to execute (19). Visual analog scales are horizontal non calibrated line ranging from minimal (0) to maximal (10) stress. A level of stress higher than 8/10 is a cut-off for stress levels requiring urgent action (20). The secondary outcomes were the sector of activity (secondary or tertiary, main sectors and other sectors studied), the working status during the lockdown [business activity ranging from decreased (0) to increased (10)], the size of the company (number of workers) and the geographical location (metropolis and countryside). Companies were classified according to the variation rate in their activity between before and during lockdown. Those with a greater than average reduction in activity were categorized as “reduced work” and the others as “continued work.”

### 2.3. Statistical analysis

Statistical analysis was performed using Stata software (version 16; StataCorp, College Station, Texas, USA). All tests were two-sided, with a Type I error set at 0.05. Categorical variables were expressed as number of subjects and associated percentages, and quantitative variables as mean ± standard deviation (especially stress level). The evolution of stress over time was evaluated using linear mixed models (for VAS of stress) or generalized linear mixed models with logit link function (for stress level >8/10). Furthermore, the stress variation between before and during lockdown was calculated (VAS during minus VAS before) and the factors associated with this variation were studied using Hedges'g effect sizes (ES). They were presented with 95% confidence intervals (CI) and interpreted according to Cohen's recommendations: 0.2 = small effect, 0.5 = medium effect and 0.8 = large effect. Finally, factors associated with stress >8/10 during lockdown were studied using logistic regressions. The results were expressed as odds ratio (OR) and 95% CI.

## 3. Results

### 3.1. Population

**Figure 1:**
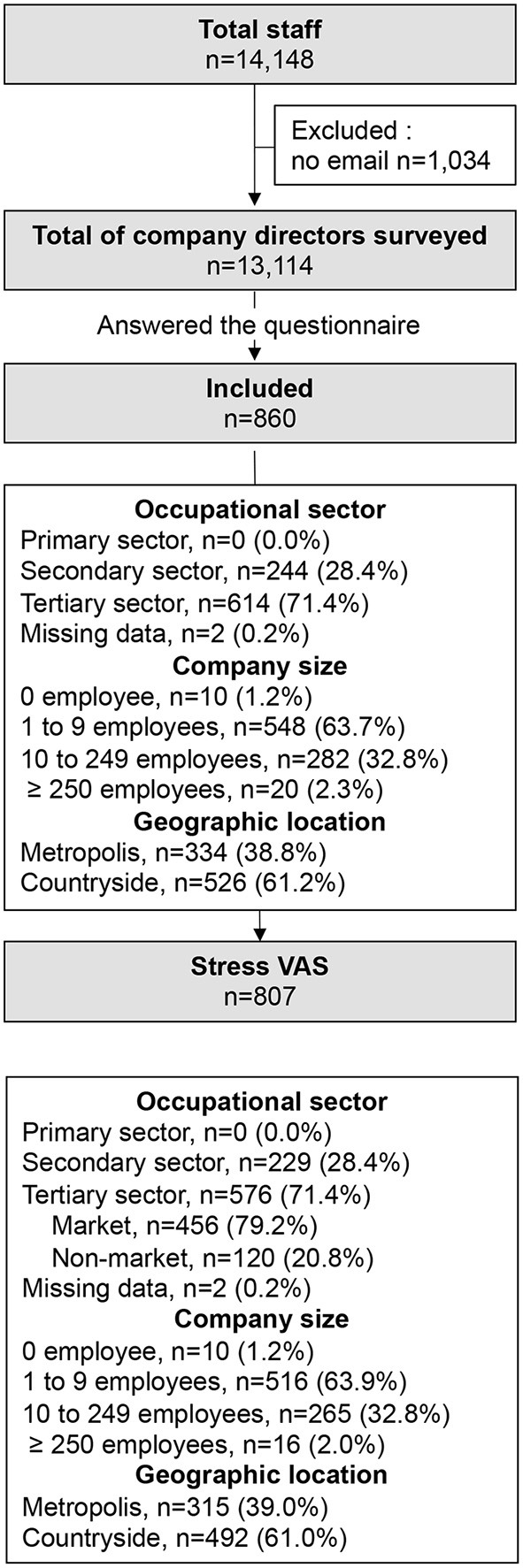
Flowchart. VAS, Visual Analog Scale.

**Figure 2:**
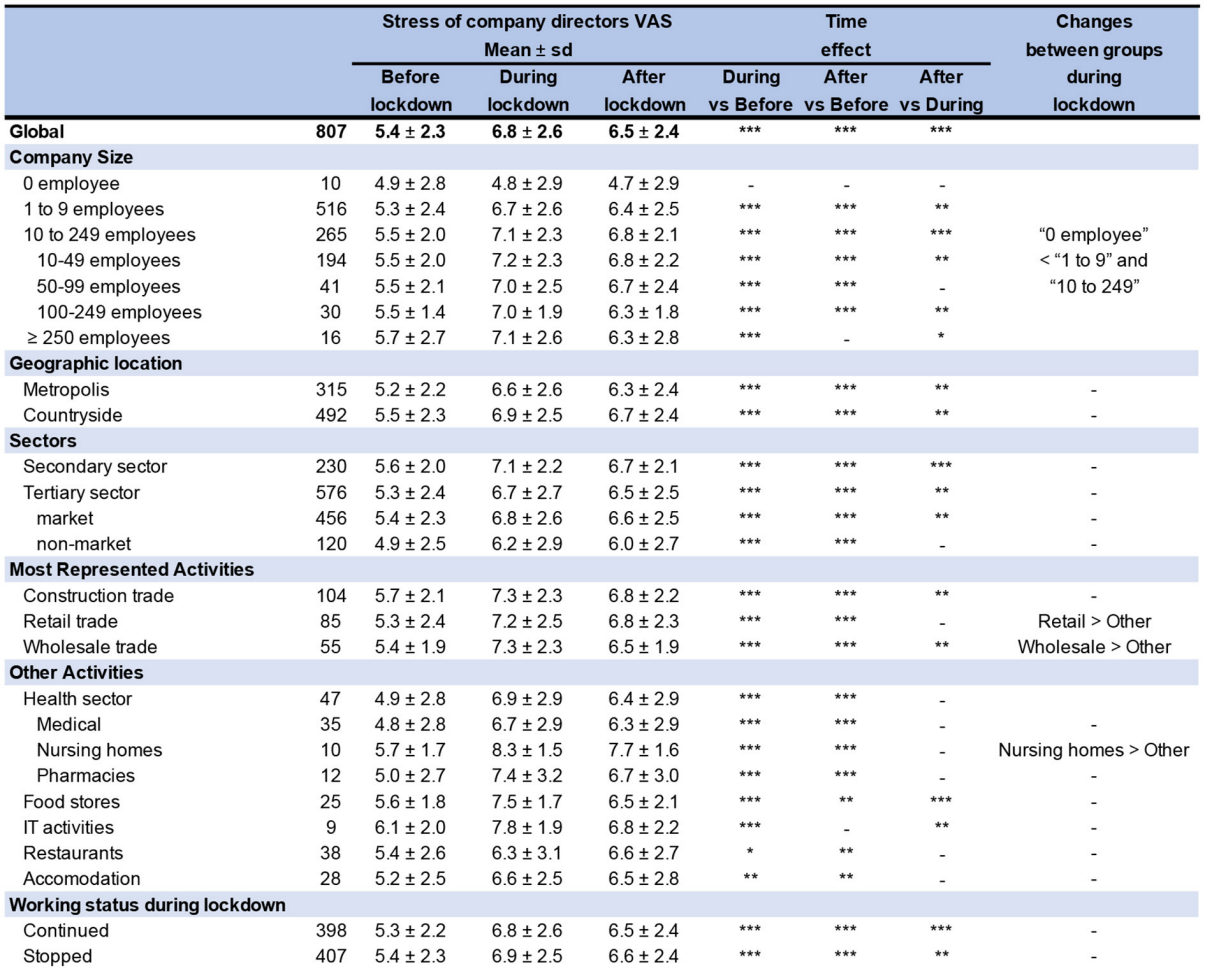
Stress of company directors. VAS, Visual Analog Scale; SD, Standard Deviation. ***p < 0.001. **p < 0.01. *p < 0.05. -: non significant.

The questionnaire was sent to the 13,114 company directors who had provided an email address (Figure 1). A total of 860 responded. Fifty-three were excluded due to incomplete data and analysis were performed on 807 (6.2%). The main sector of activity was the tertiary sector (*n* = 576, 71.4%), essentially market (*n* = 456, 56.5%). The most represented activities were construction trade (*n* = 103, 12.9%), retail trade (*n* = 85, 10.5%) and wholesale trade (*n* = 55, 6.8%). The majority of companies had one to nine employees (*n* = 516, 64.0%), or 10 to 249 employees (*n* = 265, 32.8%); with mainly companies <50 employees (*n* = 194/265, 73.2%). Companies with no employees (*n* = 10, 1.2%) and ≥ 250 (*n* = 16, 2.0%) were poorly represented. The respondent companies were mainly located in the countryside (*n* = 492, 61.0%). The business activity rate decreased by 51.7% with lockdown (5.8 ± 2.0 vs. 2.8 ± 2.8, *p* < 0.001)−49.4% (*n* = 398) were classified as continued working and 50.6% (*n* = 407) as stopped working (Figure 2). The business activity rate remained decreased during and improved after the lockdown while remaining 16.0% lower than initial (5.8 ± 2.0 vs. 4.8 ± 2.6, *p* < 0.001) (Appendix 1).

### 3.2. Stress of company directors

**Figure 3:**
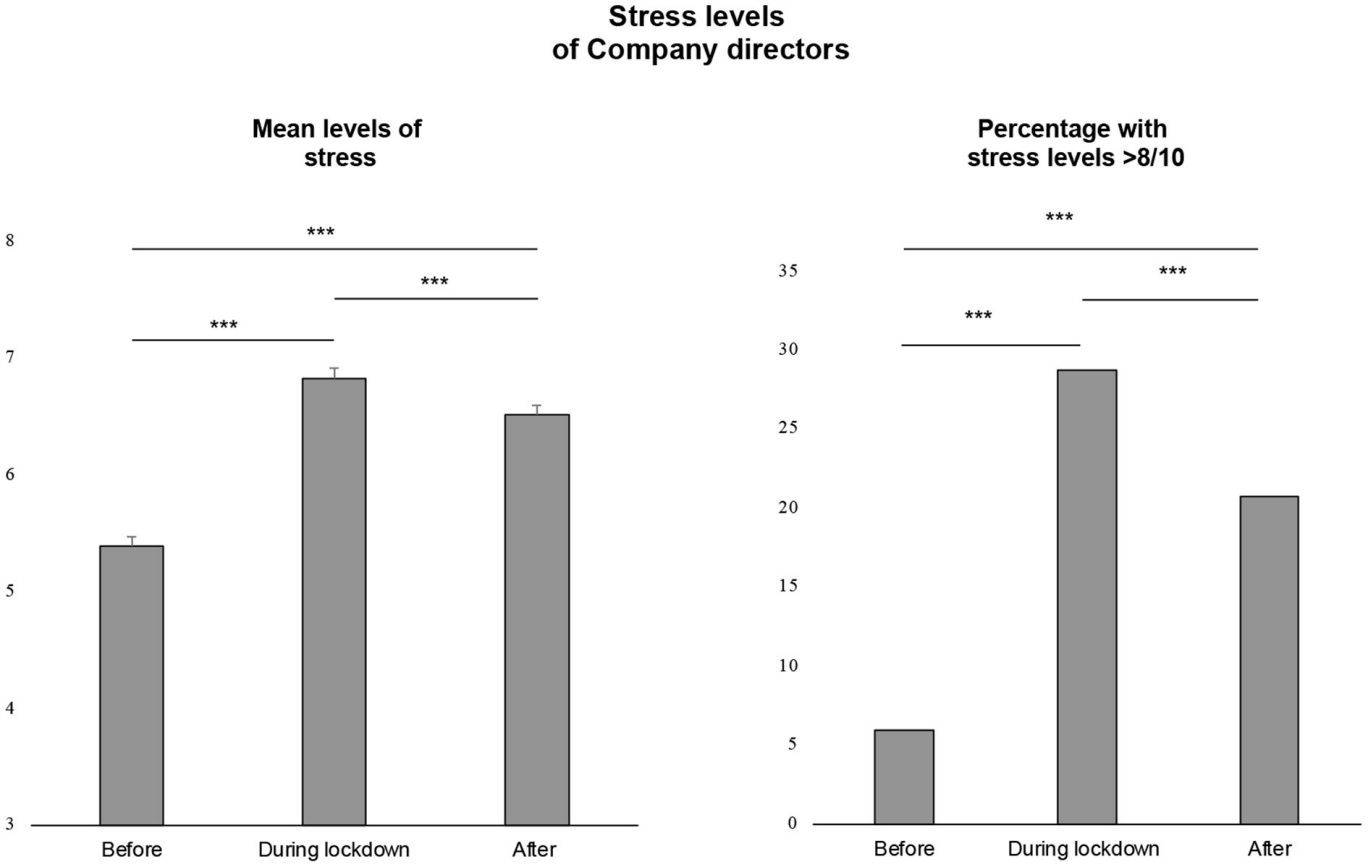
Stress levels of company directors (mean levels ± standard error of the mean and percentage with stress levels >8/10). ***: significant changes between periods (*p* < 0.001).

The level of stress at work increased by 25.9% during the first lockdown (6.8 ± 2.6 vs. 5.4 ± 2.3, *p* < 0.001) and remained 20.4% higher after (6.5 ± 2.4 vs. 5.4 ± 2.3, *p* < 0.001). The percentage of company directors with a level of stress > 8/10, intervention threshold, was 5.9% (*n* = 48) before lockdown. It increased to 28.7% (*n* = 231, *p* < 0.001) during, remaining at 20.7% after (*n* = 167, *p* < 0.001) (Figures 2, 3).

### 3.3. Influencing factors of stress of company directors

#### 3.3.1. Longitudinal analyses

Stress of company directors increased during lockdown whatever the characteristics of companies (*p* < 0.05 for stress levels before vs. first lockdown, for each variable), except for companies without employee (*n* = 10, 4.9 ± 2.8 vs. 4.8 ± 2.9, *p* = 0.57). Levels of stress remained significantly high after the lockdown except for the IT sector (6.8 ± 2.2 vs. 6.1 ± 2.0, *p* = 0.08) and companies up to 250 employees (6.3 ± 2.8 vs. 5.7 ± 2.7, *p* = 0.05). There was no significant decrease in stress after lockdown for retail trade, medical, nursing homes, pharmacies, restaurants, and accommodation sectors. Similarly, the percentage of directors with a stress > 8/10 increased with lockdown for all companies (*p* < 0.05), except for companies without employee, and for restaurants and accommodation sectors. After lockdown, the percentage of directors with a stress > 8/10 did not decrease except for construction and wholesale trade, and even increased for the accommodation sector (25.0% after vs. 14.8% during). Whatever the working status (continued or stopped) or the location (metropolis or countryside), the stress levels and the percentage of directors with stress > 8/10 increased significantly during lockdown (*p* < 0.001) without difference between groups (continued vs. stopped, or metropolis vs. countryside) (Figures 2, 3; Appendix 2).

#### 3.3.2. Effect sizes for increase in stress during lockdown

**Figure 4:**
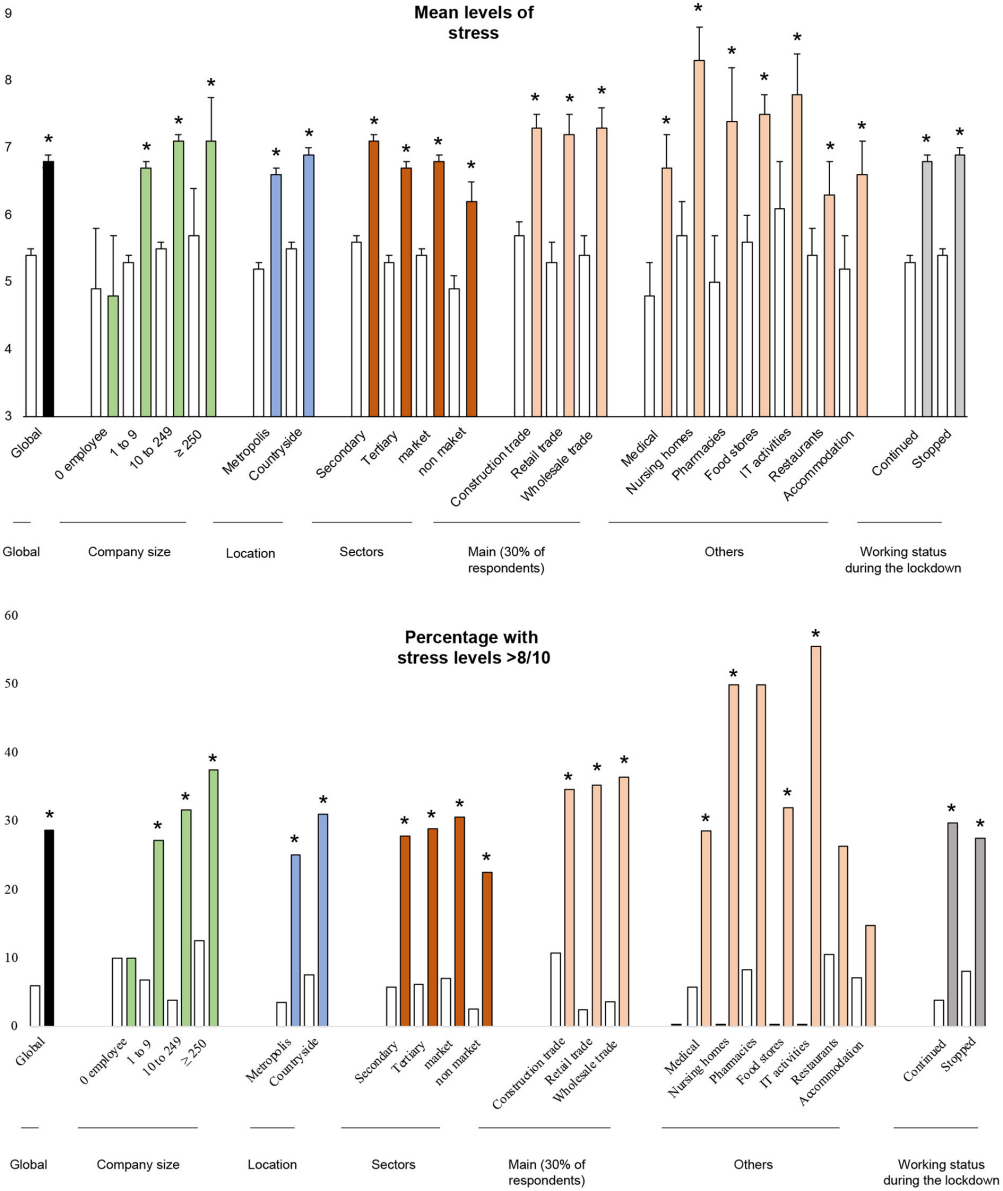
Stress before and during lockdown (mean levels ± standard error of the mean). Light shading: before the lockdown; Dark shading: during the lockdown. *: significant changes within the condition (*p* < 0.05) between before and during.

Sectors which had the biggest increase in stress levels during lockdown were retail trade (ES = 0.22, 95 CI 0.00 to 0.45), wholesale trade (0.26, 0.00 to 0.53) and nursing homes (0.55, −0.10 to 1.18). For pharmacies, this difference is at the limit of significance (0.47, −0.10 to 1.04). Biggest companies had the highest increase in stress levels (≥ 250 employees: 1.20, 0.36 to 2.03 vs. no employee; 10 to 249 employees: 0.90, 0.27 to 1.54; one to nine employees: 0.65, 0.03 to 1.28; vs. no employee). Geographical location and working status did not influence the increase of stress levels (Figure 4; Appendix 3).

#### 3.3.3. Odds ratio

**Figure 5:**
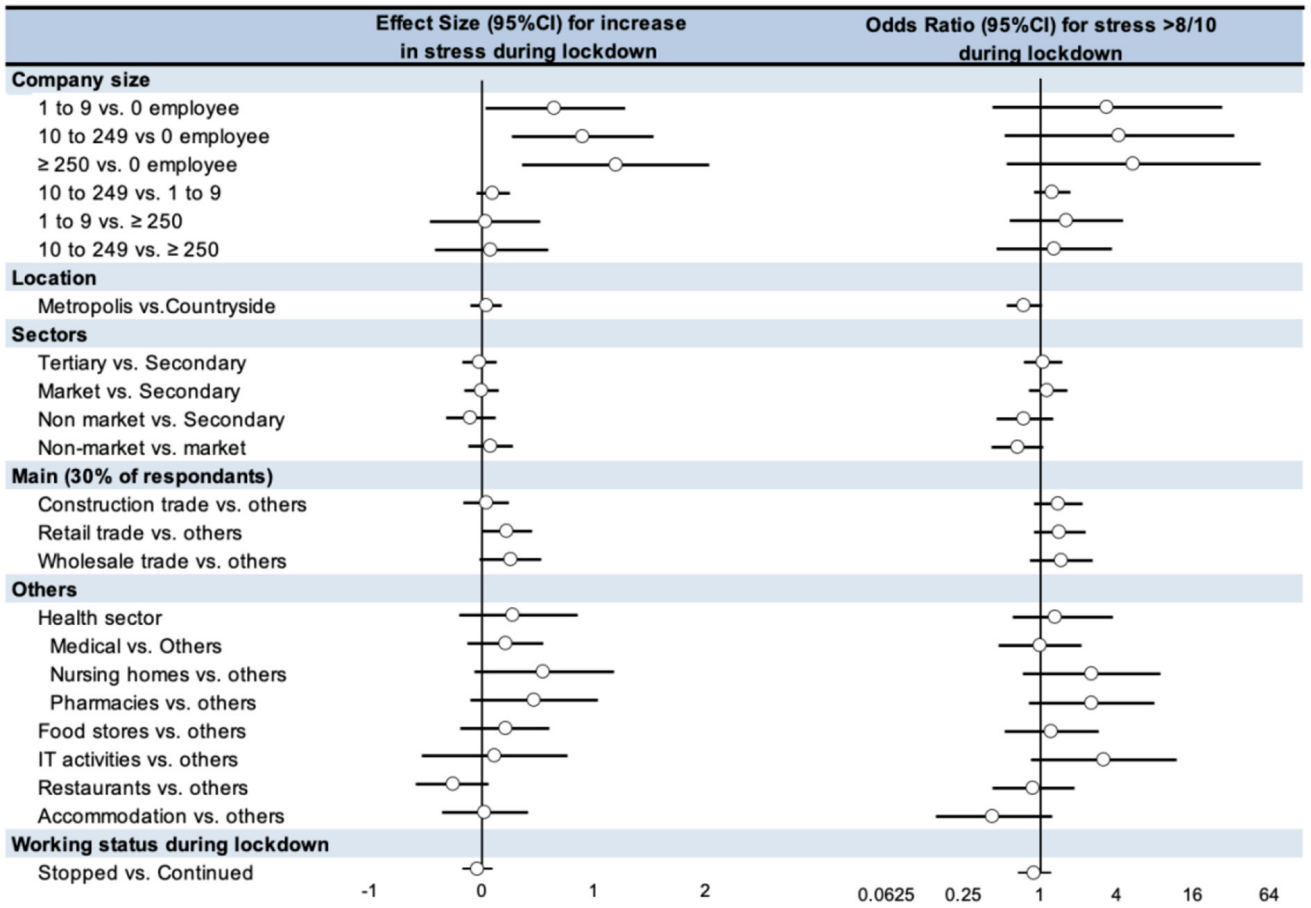
Factors influencing the increase in stress levels and risk for stress >8/10 (Odds Ratio) during lockdown. 95%CI, 95% Confidence Interval.

Sectors the most at risk of stress > 8/10 during lockdown tended to be nursing homes (OR = 2.52, 95 CI 0.73 to 8.82), pharmacies (2.54, 0.81 to 7.95), and IT activities (3.17, 0.84 to 11.90). Conversely, accommodations sector tended to have a lower risk of stress > 8/10 during lockdown (0.42, 0.15 to 1.24) followed by an increase after lockdown (25.0% after vs. 14.8% during). Biggest companies tended to have a higher risk of stress > 8/10 during the lockdown (≥ 250 employees: 5.40, 0.54 to 53.9; 10 to 249 employees: 4.18, 0.52 to 33.5; 1 to 9 employees: 3.36, 0.42 to 26.8; vs. no employee). Geographical location (metropolis vs. countryside) and working status (continued vs. stopped) did not influence the risk of stress > 8/10 (Figure 5; Appendix 4).

## 4. Discussion

The main findings were an increase of 26% in the stress of company directors during the first lockdown of the COVID-19 pandemic, and a four-time increase in the percentage of stress > 8/10 (intervention threshold). Medical and IT sectors were the most at-risk, as well as biggest companies.

### 4.1. Stress of company directors

This study demonstrated a 26% increase in stress levels of company directors, which seems higher than a 22% increase in stress levels of a general international population–also measured using visual analog scale (21). It also showed that nearly one third (29%) of company directors were at high levels of stress (> 8/10) during the lockdown. Even if threshold for high levels of stress may vary between studies, company directors seemed more at risk than prevalence of high levels of stress reported in other studies in general population: 12% in India (22), 5 and 8% in China (23), and 10% in Australia (24). Only one study reported stress levels of managers during the lockdown (25) and found lower prevalence than in the studied population. However, this study is the first study focusing specifically on the stress of company directors and reported the evolution of stress before, during, and after the first lockdown. The few studies assessing mental health of company directors during the COVID-19 pandemic were mainly not on stress but on anxiety (9), depression (9), and burnout (11), and none assessed the evolution of stress over three key periods of the pandemics. but only one assessed stress at work (12). We demonstrated a massive increase of stress of company directors. Results could be explained by the accumulation among company directors of numerous stress factors described in the literature such as gender, age, and relative income, although not assessed in our study (26, 27). It has been accentuated by the pandemic and lockdown. Indeed, they had been exposed to an overload of work and emotional requirements but also to insecurity at work (28) with difficulty in anticipating and a lack of autonomy in the face of government directives (29). Company directors had assumed strong responsibilities, including the risk of transmission of COVID-19 and the survival of their companies considering the economic difficulties (30). In addition, the stress levels of company directors before the pandemic (5.4 ± 2.3) were also superior to levels of other workers found in the literature: 4.0 ± 2.4 in French workers (20), 4.0 ± 2.7 in hospital workers (31), or 4.4 ± 2.1 managers/engineers (17), suggesting that company directors could be particularly exposed to stress like emergency health care workers (32, 33). Lastly, the stress of company managers remained high after the end of lockdown, showing the need for action to be taken by the occupational health services. Despite specific preventive strategies are needed toward the mental health of company managers, it may be difficult for company directors to reduce their stressors, especially following the massive economic impact of a global pandemic. Some authors suggested recovery strategies allowing detachment from work stressors in non-work time and advise entrepreneurs to engage in absorbing recovery activities (e.g., physical exercise, meditation, socializing) (34). However, the need for more research to better understand the unique work situation of company directors is needed. Such studies should account for several levels of analyses (within-person and between-person effects) and time perspectives (short-term, mid-term, and long-term) (34).

### 4.2. Characteristics of companies at risk of stress

The novelty of our study laid in the impact of economic sectors on stress levels of company directors during the COVID-19 pandemic. While other studies on mental health of company directors during the pandemic were more precise for sociodemographic (9–11), none evaluated the sectors of economic activity the most at-risk. Despite the global lockdown massively impacted economy (13, 14), some sectors completely stopped their activity while some other sectors were under pressure. Our study showed a higher stress and/or a trend for more severe stress for certain sectors, such as medical and IT sectors. The stress of directors of nursing homes can be explained by the high job demand and emotional overload due to the fear of the risk of contamination and the management of suffering. Indeed, health workers in charge of COVID patients are described as being at high risk of psychological consequences (35), notably in a population of nursing homes health care workers (36). Those results also showed a trend toward higher and more severe stress for the IT sector, that experienced a significant work overload due to the advent of teleworking (37) and the need to implement digital and technological tools (38). Retail and wholesale trade had also high levels of stress during lockdown, facing a huge demand (14). For the restaurant and accommodation sectors, results showed a trend toward lower stress. These two sectors have been forced to close. Being forced to close can also be extremely stressful but the impact on stress of company directors may depend from stringency of governmental lockdown and from economic public measures (9). After lockdown, the stress decreased except for the nursing homes that were still exposed, and for restaurants and accommodations for which the absence of a return to activity led to economic difficulties. Directors of large companies were more at risk of stress than directors of companies without employees. Indeed, the stress risk factors for directors differ according to the size of the company (39), particularly in terms of decision-making latitude, complexity of managing organizational change and economic survival. However, very limited literature have been published on this subject. This study did not show any difference in stress according to the location of the company, metropolis vs. countryside, which is coherent considering that the governmental directives were the same. Identifying companies the most at-risk is a necessary step to further build efficient preventive strategy (16).

### 4.3. Limitations

This study had some limitations. First of all, the sample was obtained through a voluntary online questionnaire, a method which leads to a non-response bias (40). However, despite a low response rate, we still collected an interesting sample of more than 800 company directors. In our study, this could induce a risk of non-representativity of our sample. Indeed, we had an important response rate of construction firms but a low response rate of health firms. This could be explained by the fact that directors of those firms were overwhelmed even more than usual during the lockdown contrary to the directors of constructions firms that were forced to stop all activities. The low sample in each category did not allow the odds ratio to be significant by company size and sector of activity, but we were able to differentiate several more at-risk sectors. Similarly, the effect sizes for the variation in stress during lockdown by sector of activity remained small or moderate (between 0.20 and 0.55). However, effect sizes are recommended when data are numerous to prevent from false significant findings, and we also acknowledge that none of previous studies on mental health of company directors reported effect sizes (9–11). The small sample sizes also did not allow for correlations between business activity levels over the different time periods studied and the variation in stress. Furthermore, the questionnaire was not exhaustive. Gender, age, and entrepreneurial experience of respondents were not asked to preserve anonymity. Those factors have been described as factors influencing stress (15, 41), particularly for age in a study conducted in a manager population during the COVID-19 pandemic (25). Our study also did not question the directors about the economic impact that lockdown and the pandemic may have had on their companies, that could also be stress confounders (30), such as for workload (42). In addition, 807 companies were selected solely from the population of a regional occupational health service, with no primary sector and very few large companies. Further studies should be conducted with a larger sample including primary sector and large companies on several geographical sites for generalizability. Future studies should also investigate more deeply psychosocial risk factors than we did with our single measure of stress using a single item. For example, using complementary validated questionnaires such as job-demand-control-support or effort-reward-imbalance models (41) may offer the possibility to build efficient preventive strategy, using predictive models (43, 44). Our study may have practical implications considering the putative very long-term (several years) impact of the pandemic on mental health, as we demonstrated on other population during the SARS-CoV-1 epidemic in 2003 (45).

## 5. Conclusions

The first lockdown of the COVID-19 pandemic had a major impact on the stress levels of company directors, which increased by 25.9% during lockdown. This study also demonstrated that almost a third of company directors (29%) had very high levels of stress (>8/10) during the first lockdown of COVID-19, requiring urgent action because of the risk of burnout, anxiety and depression. Furthermore, this rate was still higher after the lockdown (20.7%) compared to before lockdown (5.9%). Medical and IT sectors were particularly at risk, as well as directors of large companies. Future studies with larger sample are needed to be able to target occupational health actions among company directors the most at risk of stress. Qualitative interviews can be interesting to target precisely a potential psychosocial action. It could also be interesting to provide some longitudinal follow-up during the next months and years to assess the long-term impact of COVID-19 and lockdown.

## Data availability statement

The original contributions presented in the study are included in the article/Supplementary material, further inquiries can be directed to the corresponding author.

## Ethics statement

The study was approved by the Ethics Committee (CPP Sud-Est VI Clermont-Ferrand) and registered on ClinicalTrials.gov number NCT04308187. Written informed consent for participation was not required for this study in accordance with the national legislation and the institutional requirements.

## Author contributions

Conceptualization: CJ, MC, FD, and J-BB-M. Methodology: FD, MC, and J-BB-M. Formal analysis: CL, FD, and MC. Investigation: FD and CJ. Writing—original draft preparation, supervision, and project administration: FD. Writing—review and editing: UU, JS, GV, CL, AV, and J-BB-M. All authors have read and agreed to the published version of the manuscript.
