# Supplementary material for: Work-related stress of companies' directors during the first lockdown due to the COVID-19
Source: Front Psychiatry. 2022 Dec 22;13:975953. doi: 10.3389/fpsyt.2022.975953 (PMC9813865; doi:10.3389/fpsyt.2022.975953)
Supplement: Supplementary file 1 [file Data_Sheet_1.docx]

Supplementary Material

**Appendix 1**

Appendix 1: Evolution of business activities using visual analog scale (0 to 10). Mean levels ± Standard error of the mean.

**Appendix 2**

Appendix 2: Stress levels >8/10 (intervention threshold)

VAS : Visual Analog Scale.

***: p<0.001. **: p<0.01. *: p<0.05. - : non significant

**Appendix 3**

Appendix 3: Effect size variation of variation of mean stress levels between before and during lockdown

**Appendix 4**

# Appendix 4: Odds ratio VAS>8 during lockdown
